# Portal Vein Doppler Parameters as Indicators of Volume Status in Hemodialysis Patients Using Wireless Ultrasound

**DOI:** 10.7759/cureus.114109

**Published:** 2026-08-07

**Authors:** Shaymaa E Mohammed, Motaz Fathy, Alaa Abd el-Hamid, Mai Galal

**Affiliations:** 1 Department of Internal Medicine, Faculty of Medicine, Kasr Al-Ainy Hospital, Cairo University, Cairo, EGY

**Keywords:** egyptian patients, hemodialysis, portal vein doppler parameters, venous congestion, volume assessment, wireless ultrasound

## Abstract

Background: Accurately assessing fluid status is a critical yet challenging clinical skill. While hypovolemia marked by low blood pressure and poor tissue perfusion is a major concern, hypervolemia can also cause harmful complications. Because there is no single gold standard test, clinicians must master various non-invasive bedside tools to achieve proper fluid balance.

Objective: The objective of this study is to investigate the potential clinical value of portal vein (PV) Doppler parameters in assessing volume status and to evaluate the utility of PV Doppler parameters for assessing volume status.

Methods: A cross-sectional study was conducted on 100 Egyptian patients from a dialysis unit. All participants underwent a medical history review, clinical examination, and laboratory tests including complete blood count, liver function tests, kidney function tests, and assessment of Doppler parameters of the PV pre-dialysis and post-dialysis.

Results: The mean age of cases was 42.70 (± 12.25), while the mean age of control subjects was 45.57 (± 10.32), with no statistically significant difference in age between cases and control subjects (p-value 0.24). Female patients represented 48% and male patients 52% in cases, while in control, female subjects accounted for 46.7% and male subjects 53.3%, with no statistically significant difference between cases and control (p-value 0.898). A significant improvement was observed in the PV diameter post-dialysis (p-value 0.004) and pulsatility index (p-value < 0.001). Additionally, there was a significant improvement in the PV waveform (p-value < 0.001).

Conclusion: PV Doppler parameters reflect systemic venous congestion and changes in volume status in hemodialysis patients. Therefore, they can be used to assess pulmonary congestion in this patient population.

## Introduction

Accurate assessment and timely optimization of fluid status are essential components of critical care, particularly for patients with conditions such as heart failure, end-stage kidney disease (ESKD), and acute kidney injury (AKI) requiring dialysis [1].

Decisions regarding diuretic therapy, renal replacement therapy, and ultrafiltration goals hinge on precise evaluation of intravascular volume. The absence of a definitive gold standard often complicates clinical decision-making in this regard. Historically, clinicians have relied on patient history and physical examination to formulate differential diagnoses and identify the most likely underlying cause. However, methodological limitations in the supporting studies including small sample sizes, questionable comparison groups, and limited generalizability often compromise the reliability and accuracy of these traditional methods. While physical examination remains an important tool, its subjective nature can introduce bias into the decision-making process. While thorough clinical examination remains crucial, its subjective nature can sometimes introduce bias into decision-making processes. To enhance the precision of assessments, modern medicine has integrated various tools and techniques, including advanced imaging modalities and invasive monitoring methods like central venous pressure (CVP) and pulmonary artery catheter wedge pressure monitoring. In recent years, point-of-care ultrasound (POCUS) has emerged as a valuable non-invasive tool, gaining widespread adoption as the "fifth vital sign" in clinical medicine [2].

Recent advancements in bedside ultrasound technology have enabled the evaluation of organ congestion and its severity by analyzing venous flow patterns in the liver or kidneys using Doppler ultrasonography [3]. Changes in renal and portal venous flow patterns have been shown to correlate well with each other and are consistently associated with organ injury due to congestion [4,5].

Recent studies have suggested that integrating B-mode ultrasound of the inferior vena cava with Doppler assessment of the portal, hepatic, and intrarenal veins enhances the accuracy of evaluating organ venous congestion [6].

Venous Doppler ultrasound may also serve as a valuable tool for monitoring decongestion induced by removing fluid. Experimental volume expansion in individuals with heart failure has been shown to worsen venous Doppler patterns [7], whereas volume removal through diuretic therapy improves these patterns [8]. Considering the high prevalence of right heart failure and pulmonary hypertension (>60%) among ESKD patients undergoing hemodialysis [9], accurate evaluation of systemic venous congestion and volume status in this population is critical.

The purpose of the present study is to provide a noninvasive method to monitor the effectiveness of decongestive therapy and diuretic use, and also to guide the amount of ultrafiltration in hemodialysis. We study the potential clinical value of portal vein (PV) Doppler parameters in predicting the presence of venous congestion and personalizing ultrafiltration strategies in hemodialysis to decrease the incidence of intradialytic hypotension and prevent the deleterious effects of chronic congestion in intra-abdominal organs. The clinical utility of point-of-care venous Doppler monitoring has recently expanded beyond simple prognosis to active therapeutic guidance. Notably, Abu-Naeima et al. [10] demonstrated that severe systemic venous congestion, quantified via the Venous Excess Ultrasound (VExUS) grading system and the renal venous stasis index (RVSI), was independently associated with reduced loop diuretic efficiency and worsening renal function in acute cardiorenal syndrome patients managed at our institution. While their findings underscore the impact of venous congestion on pharmacological decongestion patterns, the dynamic utility of handheld wireless ultrasound specifically for monitoring mechanical fluid removal such as ultrafiltration tailoring in chronic hemodialysis patients remains to be fully elucidated [10].

Specifically, a significant knowledge gap exists regarding how these venous Doppler parameters behave during active ultrafiltration in patients undergoing hemodialysis, who experience rapid and massive fluid shifts. Moreover, while standard tethered ultrasound machines are bulky and impractical in cramped dialysis units, the clinical utility of handheld wireless ultrasound for real-time bedside monitoring of mechanical volume removal has not been fully elucidated.

To address these gaps, the purpose of this study is to evaluate the clinical value of PV Doppler parameters using handheld wireless ultrasound to estimate and monitor volume status in hemodialysis patients. By tracking dynamic venous flow variations, we aim to provide an objective, bedside approach to tailor ultrafiltration strategies, ultimately aiming to prevent the deleterious effects of intradialytic hypotension and fluid overload.

## Materials and methods

Subjects and methods

This observational cross-sectional study involved 100 patients undergoing hemodialysis and 30 healthy control subjects aged 20-60 years. Participants were recruited from the internal medicine department and dialysis units at Kasr Al-Ainy Hospital, Cairo University. The primary purpose of including a healthy control group in this study is twofold: (i) To establish local baseline reference values: Since we utilized a handheld wireless ultrasound device, it was essential to establish standard baseline reference parameters (including normal PV diameter, pulsatility index (PI), and continuous waveform patterns) within a healthy Egyptian cohort for accurate comparison; (ii) To demonstrate the extent of systemic venous congestion: By comparing pre-dialysis ESKD patients directly against healthy individuals, we were able to statistically confirm the baseline presence of significant fluid overload and venous congestion in the dialysis cohort prior to ultrafiltration. 

Ethical approval was granted by the Faculty of Medicine, Cairo University, and all participants or their legal guardians provided informed consent. The inclusion criteria required hemodialysis patients whose blood pressure was controlled, while exclusion criteria included individuals with metastatic cancer, a history of acute coronary syndrome or heart failure, uncontrolled hypertension, chronic liver or lung disease, morbid obesity, or systemic hematological disorders. 

Patients with a known history of chronic heart failure, pulmonary hypertension, or advanced chronic lung disease were intentionally excluded from this study. This rigorous restriction was implemented to isolate the direct effects of intravascular volume overload and subsequent ultrafiltration on PV hemodynamics, thereby removing the confounding influence of baseline structural right heart disease or fixed cardiac pressure transmission on the venous system.

Imaging assessment

Doppler Assessment 

To minimize operator-dependent bias and ensure measurement reproducibility, all wireless handheld ultrasound examinations were performed by a single investigator who underwent standardized training in POCUS and venous Doppler acquisition. To determine intra-observer reliability, a subset of 15 randomly selected participants underwent repeat PV assessments 15 minutes apart under identical baseline conditions. The intra-class correlation coefficient (ICC) for the PV diameter and the PV PI was calculated, demonstrating excellent reproducibility (ICC > 0.85)."

To eliminate confounding hemodynamic variations, all Doppler waveforms and measurements were strictly acquired at the end-expiration phase during a brief, unforced breath-hold. Furthermore, all participants were maintained in a fasting state for at least four hours prior to their pre-dialysis ultrasound evaluation to prevent postprandial splanchnic hyperemia from falsely altering baseline PV flow patterns and pulsatility fractions.

**Figure 1:**
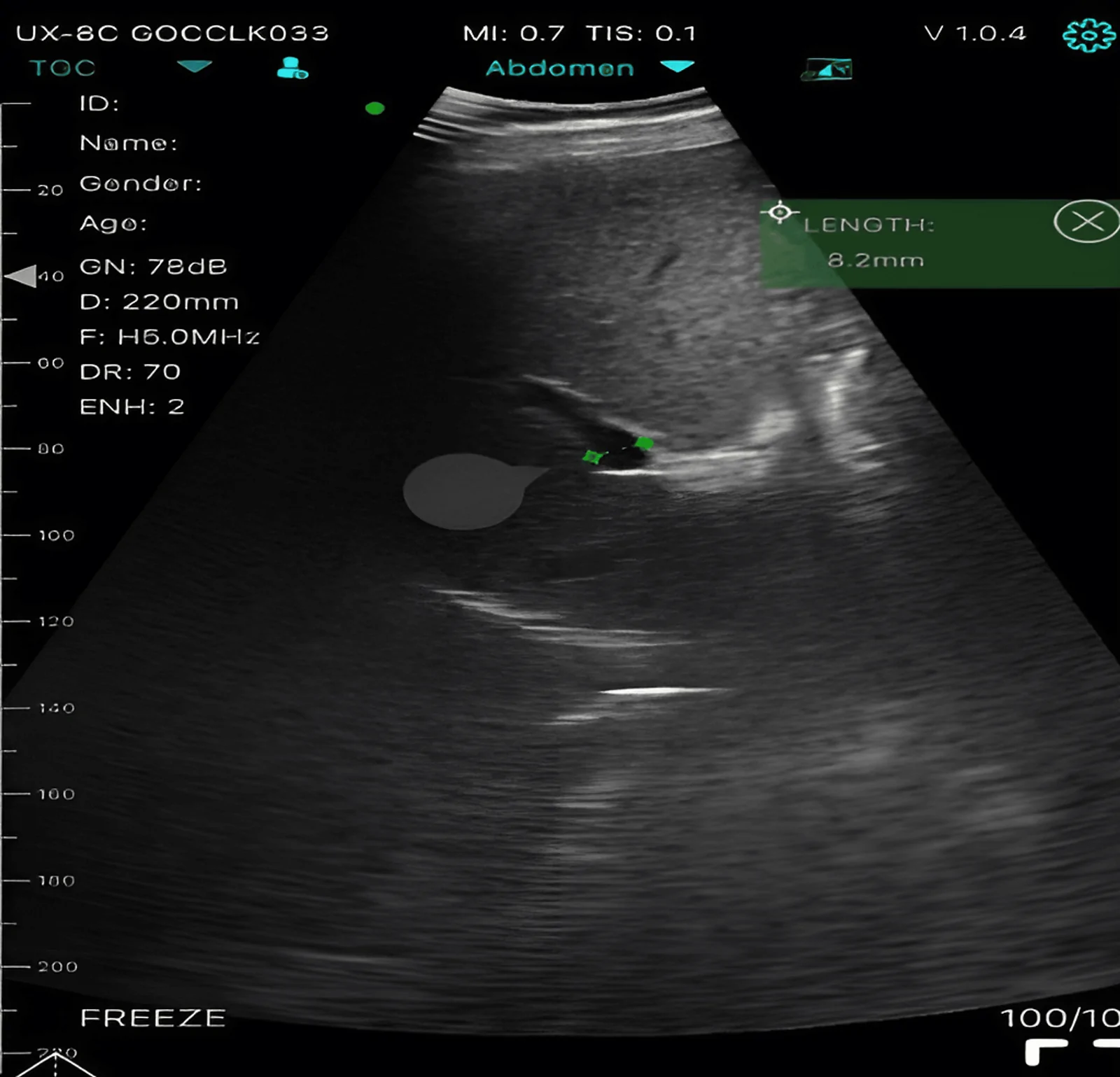
Assessment of the portal vein diameter by wireless handheld ultrasound

PV images were acquired in the supine position using a curved probe of a wireless handheld US (UX-8C GOCCLK033; Clarius Mobile Health, Vancouver, Canada) placed in the right intercostal space. The wireless handheld ultrasound system (UX-8C GOCCLK033) was equipped with a 3.5-5.0 MHz convex transducer. The system provides B-mode imaging for anatomical localization, Color Doppler for vessel identification and flow assessment, and pulsed-wave Doppler for spectral waveform analysis and velocity measurements. The PV diameter was measured in the posterior-axillary coronal view between the ninth and eleventh intercostal spaces (Figure 1).

**Figure 2:**
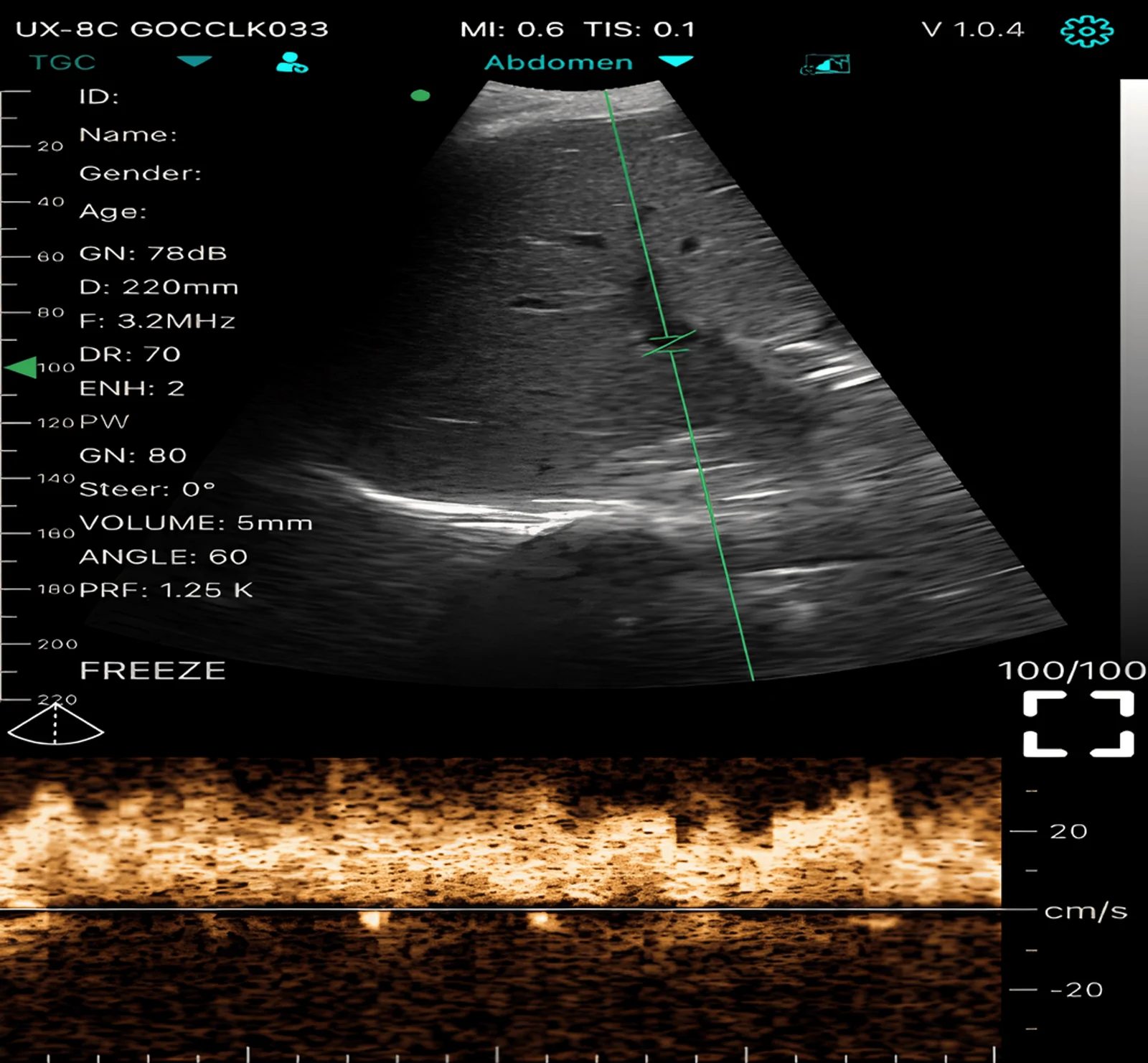
Assessment of the portal vein waveform by wireless handheld ultrasound

The spectral wave pattern was evaluated in the posterior-axillary coronal view between the ninth and eleventh intercostal spaces (Figure 2).

The PV pulsatile index was calculated as: $\text{PV Pulsatile Index (\%)} = \frac{\mathrm{V}_{\mathrm{max}} - \mathrm{V}_{\mathrm{min}}}{\mathrm{V}_{\mathrm{max}}} \times 100$

Where Vmax is the peak systolic velocity and Vmin is the minimum diastolic velocity.

This index quantifies the flow variation through the cardiac cycle. 

The PV Doppler waveforms were quantitatively assessed and classified into normal (<30% pulsatility), mild/moderate (30-49% pulsatility), and severe (≥50% pulsatility) grades. This classification follows the validated VExUS protocol for assessing venous congestion, as described by Beaubien-Souligny et al. [6].

Statistical methods

Data were coded and entered using IBM SPSS Statistics for Windows, Version 28 (Released 2021; IBM Corp., Armonk, New York, United States). Data were summarized using mean and standard deviation for quantitative variables and frequencies (number of cases) and relative frequencies (percentages) for categorical variables. Comparisons between groups were done using an unpaired t-test for normally distributed quantitative variables, while a non-parametric Mann-Whitney test was used for non-normally distributed quantitative variables. For comparison of serial measurements within each patient (pre and post), a paired t-test was used in normally distributed quantitative variables, while a non-parametric Wilcoxon signed rank test was used for non-normally distributed quantitative variables.

For comparing categorical data, the chi-square (χ²) test was performed. Exact test was used instead when the expected frequency was less than 5. For comparison of serial measurements within each patient, the non-parametric McNemar test and marginal homogeneity test were used.

Correlations between quantitative variables were done using Spearman's correlation coefficient. P-values less than 0.05 were considered statistically significant.

## Results

The present study included 100 Egyptian patients from dialysis units at Kasr Al-Ainy Hospital who met the inclusion and exclusion criteria, as well as 30 Egyptian subjects as a control group. Female patients represented 48% (n = 48) and male patients 52% (n =52), while in the control group, female subjects were 46.7% (n=14) and male subjects 53.3% (n= 16). There was no statistically significant difference in gender distribution between the cases and controls (P-value 0.898) (Table 1).

**Table 1:** Distribution of gender between cases and controls

		Cases		Controls		P-value
		Count	%	Count	%	
Gender	Male	52	52.0%	16	53.3%	0.898
	Female	48	48.0%	14	46.7%	

The mean age of cases was 42.70 (± 12.25), while the mean age of control subjects was 45.57 (± 10.32), with no statistically significant difference in age between cases and control (P-value 0.24). Mean BMI in cases was 27.38 ± 4.29 kg/m^2^ and that in control was 26.92 ± 4.97 kg/m^2,^ with no statistically significant difference between cases and control (P-value 0.624) (Table 2).

**Table 2:** Distribution of age and BMI between cases and controls

	Cases		Controls		P-value
	Mean	SD	Mean	SD	
Age	42.70	12.25	45.57	10.32	0.247
BMI	27.38	4.29	26.92	4.97	0.624

Comparison parameters between Doppler data of cases and control 

The mean PV diameter in pre-dialysis cases was 1.17 (±0.41), which was significantly greater than that of the control subjects (0.90 ± 0.25) with a P-value of < 0.001. Similarly, the mean PV PI in pre-dialysis cases was 41.98% (±14.45), markedly higher than that of the control group, which was 24.83% ±4.04 also with a P-value of < 0.001 (Table 3).

**Table 3:** The difference in Doppler parameters of the portal vein between cases and controls PV: Portal vein; PI: pulsatility index

	Cases		Controls		P-value
	Mean	SD	Mean	SD	
PV diameter pre-dialysis	1.17	0.41	0.90	0.25	< 0.001
PV PI pre-dialysis (%)	41.98	14.45	24.83	4.04	< 0.001

Regarding the Doppler waveform of the PV, there was a statistically significant difference between cases and controls (P < 0.001) (Table 4).

**Table 4:** The difference in the Doppler waveform of the portal vein between cases and controls PV: Portal vein

		Cases		Controls		P-value
		Count	%	Count	%	
PV wave pre-dialysis	Normal	44	44.0%	30	100.0%	< 0.001
	Abnormal	56	56.0%	0	0.0%	

Comparison between portal vein Doppler parameters pre- and post-dialysis

Considering the PV diameter, the mean PV diameter pre-dialysis in the cases was 1.17 ± 0.41 cm, which was statistically significantly higher than the mean PV diameter post-dialysis, which was 1.15 ± 0.39 cm with a P-value of 0.004. Similarly, the mean PV PI pre-dialysis in the cases was 41.98 ± 14.45, which was statistically significantly higher than the PV PI post-dialysis (27 ± 9.43) with a P-value < 0.001 (Table 3). This means that PV diameter and PI can identify systemic venous congestion and its improvement through hemodialysis (Table 5).

**Table 5:** The difference in portal vein Doppler parameters pre-dialysis and post-dialysis PV: Portal vein; PI: pulsatility index

	Cases		P-value
	Mean	SD	
PV diameter pre-dialysis	1.17	0.41	0.004
PV diameter post-dialysis	1.15	0.39	
PV PI pre-dialysis (%)	41.98	14.45	< 0.001
PV PI post-dialysis (%)	27.00	9.43	

The post-dialysis ultrasound examination was performed within (15 to 30 minutes) after the completion of the hemodialysis session, once the patient was hemodynamically stable.

Comparison between waveforms of PV pre-dialysis and post-dialysis in cases

Table 6 shows comparison between waveforms of PV pre-dialysis and post-dialysis in cases. A statistically significant correlation was observed in PV waveform changes before and after dialysis. The percentage of normal waveforms increased from 44% (44 patients) pre-dialysis to 88% (88 patients) post-dialysis, while the percentage of abnormal waveforms decreased from 56% (56 patients) to 12% (12 patients) post-dialysis with a P-value of < 0.001 (Table 6). 

**Table 6:** Comparison of waveforms of PV pre-dialysis and post-dialysis in cases PV: Portal vein

Cases		Pre		Post		P-value
		Count	%	Count	%	
PV wave	Normal	44	44.0%	88	88.0%	< 0.001
	abnormal	56	56.0%	12	12.0%	
	Mild	16	16.0%	28	28.0%	
	Moderate	36	36.0%	30	30.0%	
	Severe	26	26.0%	0	0%	

Relation between the amount of ultrafiltration and change in Doppler parameters in hemodialysis

Table 7 shows the relation between the amount of ultrafiltration and change in Doppler parameters in hemodialysis. A statistically significant negative correlation was observed between the amount of ultrafiltration and changes in PV diameter (r = -0.481, P < 0.002) as well as PV PI (r = -0.842, P < 0.001). These findings suggest that monitoring PV diameter and PI can provide valuable guidance for determining the appropriate amount of ultrafiltration in patients undergoing hemodialysis (Table 7).

**Table 7:** The relation between the amount of ultrafiltration and change in Doppler parameters in hemodialysis PV: Portal vein; PI: pulsatility index

	Amount of Ultrafiltration		
	Correlation Coefficient	P-value	N
PV diameter change	-0.481	0.002	100
PV PI change	-0.842	< 0.001	100

## Discussion

Evaluating fluid and hemodynamic status is a fundamental skill for nephrologists, crucial for a wide range of consultations, from hypertension and electrolyte imbalances to the management of AKI and ESKD. Historically, physical examination findings such as jugular venous distention, rales, peripheral edema, and third heart sounds have been relied upon to assess fluid status. While these signs can be helpful in severe cases, they are often insensitive for detecting subtle volume overload [11].

Our study was similar to that of Tonelli et al., which revealed a high prevalence of venous congestion in hospitalized ESKD patients on hemodialysis, with 88% showing abnormal Inferior vena cava diameter (IVCd) and 63% altered PV pulsatility fraction (PVPF). Many were admitted for volume overload due to missed dialysis or heart failure. It is the first prospective study demonstrating VExUS improvement after mechanical fluid removal. A single hemodialysis session with ~2,000 mL ultrafiltration reduced PVPF by 44%, significantly more than IVCd (16%), while hepatic vein Doppler (HVD) did not show significant improvement. PVPF emerged as a more sensitive marker of venous decongestion compared to other ultrasonographic parameters [12].

Our results showing rapid normalization of PV flow indices post-dialysis strongly complement the acute care data reported by Abu-Naeima et al. While they demonstrated that a failure to clear venous congestion over 72 hours was strongly linked to persistent renal dysfunction and diuretic resistance, our data prove that mechanical ultrafiltration can achieve rapid, session-specific decompression of the splanchnic venous bed. This is reflected in the dramatic shift from abnormal to normal portal venous waveforms post-dialysis (56% down to 12%, P < 0.001) observed in our cohort [10].

Recent research by Argaiz et al. has demonstrated that diuretic-induced decongestion in cardiorenal syndrome can lead to a complete resolution of PV flow alterations, even in the presence of a persistently dilated inferior vena cava (IVC) in patients with predominantly right-sided heart failure [13].

Guinot et al. demonstrated that the PV PI was the most reliable predictor of a positive response to diuretic therapy in patients admitted to a cardiac care unit. In contrast, neither IVC nor hepatic vein (HV) Doppler parameters were found to be adequate predictors of diuretic responsiveness. The authors suggest that alterations in HV Doppler may be more reflective of underlying right heart dysfunction than changes in volume status. For instance, a significant alteration in HV Doppler, such as a reversed S wave, can indicate severe tricuspid regurgitation, independent of fluid overload [14].

Further supporting this framework, a prospective trial by Beaubien et al. evaluated VExUS in critically ill patients with AKI. Both HVD and pulmonary venous distension (PVD) were linked to underlying right heart dysfunction. However, only PVD correlated with cumulative fluid balance and demonstrated improvement after 72 hours [15]. Additionally, Aslaner et al. found that PVD, but not IVC or HVD, was associated with the need for urgent hemodialysis due to volume overload. As changes in venous congestion were assessed after a single intradialytic hypervolemia session, it's possible that insufficient volume was removed to significantly impact IVC or HVD, while PVD alterations responded more rapidly [16].

Accordingly, in our study, the probability of having an altered PVD (decreased) increased following ultrafiltration. Our findings suggest that changes in PV diameter and PI may serve as valuable indicators for guiding the amount of ultrafiltration in patients undergoing hemodialysis.

Previous studies have demonstrated that PVD can rapidly detect changes in hemodynamics, even within seconds of manually compressing a high-flow arteriovenous fistula [17]. It is plausible that further volume removal, once PVD abnormalities have resolved, could lead to a more significant reduction in IVC diameter.

Interestingly, a pilot study in ESKD patients demonstrated that changes in IVC diameter more accurately reflected changes in volume status compared to measured venous pressures [18]. This suggests that alterations in vascular compliance (the ratio of volume change to pressure change) occur more rapidly than changes in central pressures. With PVPF, being highly reliant on systemic venous compliance, Argaiz et al. provide a plausible explanation for its rapid improvement with volume removal. The amount of fluid removed did not correlate with improvements in any of the VExUS parameters. This is unsurprising, as venous congestion is only one of multiple compartments where fluid can accumulate [13]. The distribution of fluid among vascular, tissue, and interstitial compartments, as well as dynamic changes in underlying cardiovascular function, introduce variability in the response to fluid removal. A significant increase in the prevalence of right ventricular dilation and dysfunction has been observed following arteriovenous fistula creation [19].

This rapid physiological shift highlights the importance of individualizing decongestive targets. As observed by Abu-Naeima et al., traditional clinical metrics like body weight changes, fluid balance tracking, and CVP are often imprecise proxies for organ-specific congestion. In their acute cohort, relative changes in PV pulsatility fraction and RVSI responded far more dynamically to fluid shifts than absolute changes in CVP. Our study mirrors this physiological principle in a chronic setting; the significant negative correlation we found between ultrafiltration volume and changes in PV Doppler parameters highlights wireless ultrasound as a sensitive tool capable of catching real-time vascular compliance changes that standard clinical parameters miss [10].

The pilot study is comparable to that by Andrei et al. This prospective observational study assessed VExUS scores and hemodynamic parameters in ICU patients at four time points during their stay. Among 145 patients, moderate (16%) and severe (6%) venous congestion were infrequent and stable throughout the study. No significant association was found between admission VExUS scores and AKI (p = 0.136) or 28-day mortality (p = 0.594). Admission VExUS ≥ 2 did not predict AKI or mortality, with consistent findings at subsequent time points. The study concluded that early VExUS assessment had limited prognostic value for systemic venous congestion in this ICU population [20].

This study has several limitations. Firstly, it is a small, observational, cross-sectional study involving randomly selected kidney disease patients admitted to a single center for a single hemodialysis session with varying degrees of volume status. We did not follow up with subsequent dialysis sessions to assess further decongestion. Additionally, liver diseases, such as steatosis, can reduce PV pulsatility by shielding PV from cardiac pressure transmission. Furthermore, young, healthy individuals, particularly those with a low body mass index, may exhibit variable degrees of pulsatility. Additionally, certain baseline clinical characteristics, such as dialysis vintage, Kt/V adequacy, ultrafiltration volume, and primary kidney disease etiologies, were not captured during the initial data collection, which represents a limitation of this study.

## Conclusions

PV Doppler ultrasound is a simple, low-cost technique that can be readily used at the bedside to assess volume status before and after dialysis sessions. The current study demonstrates that PV Doppler parameters are significantly associated with changes in volume status following hemodialysis, highlighting its potential utility in evaluating fluid shifts. Future prospective cohort studies with larger sample sizes and longer follow-up periods are recommended to further investigate its role in clinical fluid management and to validate its feasibility prior to broader integration into routine dialysis protocols.
